# Calcium Activity of Upper Thoracic Dorsal Root Ganglion Neurons in Zucker Diabetic Fatty Rats

**DOI:** 10.1155/2013/532850

**Published:** 2013-04-14

**Authors:** Marie Louise Ghorbani, Niels C. B. Nyborg, Bjarne Fjalland, Majid Sheykhzade

**Affiliations:** ^1^Department of Drug Design and Pharmacology, Faculty of Health and Medical Sciences, University of Copenhagen, 2100 Copenhagen, Denmark; ^2^Non-Clinical Development, Novo Nordisk A/S, 2760 Maaloev, Denmark

## Abstract

The aim of the present study was to examine the calcium activity of C_8_-T_5_ dorsal root ganglion (DRG) neurons from Zucker diabetic fatty rats. In total, 8 diabetic ZDF fatty animals and 8 age-matched control ZDF lean rats were employed in the study. C_8_-T_5_ dorsal root ganglia were isolated bilaterally from 14 to 18 weeks old rats, and a primary culture was prepared. Calcium activity was measured ratiometrically using the fluorescent Ca^2+^-indicator Fura-2 acetoxymethyl ester. All neurons were stimulated twice with 20 mM K^+^, followed by stimulation with either 0.3 or 0.5 **μ**M Capsaicin, alone or in combination with algogenic chemicals (bradykinin, serotonin, prostaglandin E2 (all 10^−5^ M), and adenosine (10^−3^ M)) at pH 7.4 and 6.0. Neurons from diabetic animals exhibited an overall increased response to stimulation with 20 mM K^+^ compared to neurons from control. Stimulation with Capsaicin alone caused an augmented response in neurons from diabetic animals compared to control animals. When stimulated with a combination of Capsaicin and algogenic chemicals, no differences between the two groups of neurons were measured, neither at pH 7.4 nor 6.0. In conclusion, diabetes-induced alterations in calcium activity of the DRG neurons were found, potentially indicating altered neuronal responses during myocardial ischemia.

## 1. Introduction

It is estimated that 366 million people worldwide in the age of 20–79 years were affected by diabetes in 2010 and the prevalence is expected to increase to 552 million people in 2030 [1]. Cardiovascular disease is the main cause of death among people with diabetes, accounting for 50% of all diabetic deaths [1]. Diabetes mellitus is per se a risk factor for developing cardiovascular disease, with ischemic heart disease being the most frequently found [2], but also diabetic neuropathy, specifically cardiovascular autonomic neuropathy, is considered a risk factor [3–6]. Silent myocardial ischemia and infarction as well as sudden death are also more frequently found in patients where diabetes and cardiovascular autonomic neuropathy coexist and cardiovascular autonomic neuropathy is furthermore believed to be a contributing factor to the higher mortality and morbidity found in patients with diabetes mellitus following myocardial ischemia or infarction [3–7].

Neurons that can contribute to the pathological picture of autonomic neuropathy are the afferent sensory nerves coming from the organ, the neurons involved in central processing of the nociceptive signal, and the efferent autonomic nerves leading to the organ and regulating its function [8]. 

During myocardial ischemia, lactic acid is produced, causing a decrease in local pH (decrease of epicardial pH from 7.35 to 6.98 after 5 min of coronary artery occlusion has been demonstrated) [9], and release of inflammatory and/or algogenic (i.e., pain-causing) mediators, such as bradykinin, adenosine, serotonin, prostaglandin E_2_, and histamine [10]. These metabolic components have been shown to activate/sensitize spinal and vagal afferents in the heart. The effect is (1) release of neuropeptides leading to local effects in the myocardium and (2) transmission of the nociceptive signal through the primary afferents (via the dorsal root ganglia (DRGs)) to segments C_8_-T_9_ in the spinal cord [11–15] or to the nucleus of the solitary tract in medulla oblongata. Neurons can project to the brain giving rise to perception of anginal pain and associated feelings (e.g., nausea and anxiety) and/or activate autonomic efferent fibers, leading to regulation of cardiovascular function [11].

Diabetes-induced alterations in cardiac sensory nerves could thus have pronounced effects on the way a patient responds to myocardial ischemia and infarction, including changes in perception of the pain often accompanying myocardial ischemia and infarction, and in the cardiac compensatory responses. 

Diabetic neuropathy is thought to be caused by several hyperglycemia-induced changes: neurovascular dysfunction, lack of trophic support, and metabolic changes, such as an increased polyol pathway flux, increased production of advanced glycation end products (AGEs), and oxidative stress [16]. Furthermore, alterations in [Ca^2+^]_i_ homeostasis have been suggested to contribute to the pathogenesis of diabetic neuropathy [16–18]. Altered calcium channel activity has been shown in primary [18] and secondary sensory neurons from diabetic rodents [19].

Very little is known of the consequences of diabetes on sensory nerve function of primary afferents innervating the heart. Schultz found a decreased activity of cardiac vagal afferents in STZ-diabetic rats compared to control rats after epicardial application of bradykinin and capsaicin [20] and recently, diabetes-induced alterations in the spinal processing of nociceptive input from the heart were found in diabetic rats following cardiac stimulation [21].

The purpose of the present study was to investigate the calcium activity of C_8_-T_5_ dorsal root ganglion (DRG) neurons (primary neuron) in Zucker diabetic fatty rats, a spontaneous animal model for type 2 diabetes, and the effect of stimulation with algogenic substances, known to play part during myocardial ischemia, as well as with capsaicin, the pungent ingredient in chili peppers, at pH 7.4 and 6.0. To the best of our knowledge, this is the first study assessing the calcium activity in upper thoracic DRG neurons from any type of diabetic rat.

## 2. Methods and Materials

### 2.1. Animals

Animal procedures were approved by the Danish Animal Inspectorate (license no. 2004/561-943). 

Experiments were performed in male diabetic Zucker diabetic fatty (ZDF) rats as well as age-matched nondiabetic ZDF rats. The ZDF rat is a spontaneous animal model for type 2 diabetes in which the animals are homozygous for nonfunctional leptin receptors [22, 23]. On a Purina 5008 diet, hyperglycemia will develop as early as an age of approximately 7 weeks [23].

The animals were stabled in pairs and were fed with Purina 5008, an energy-rich fodder. Once a week, the animals were weighed and blood glucose was measured from a tail vein using a glucometer (OneTouch Ultra2, Lifescan).

### 2.2. Primary Culture

At the age of 14–18 weeks, the rats were decapitated and C_8_-T_5_ DRG's were removed bilaterally and placed in Hank's Buffered Salt Solution (HBSS).

The ganglia were transferred to 5 mL papain solution (2 mg/mL, Sigma-Aldrich) for 35 min at 37°C. After centrifugation (2800 rpm, 2 min) the supernatant was removed and the pellet was resuspended in 5 mL of a collagenase/dispase solution (Collagenase: 1.11 mg/mL, Medinova; Dispase: 1.33 mg/mL, Roche Diagnostics GmbH). The solution was kept at 37°C for 20 min with routinely mixing. After centrifugation (2800 rpm, 2 min) and removal of supernatant, the cells were triturated in 1 mL HBSS (by at least 10 passages through a fire-polished Pasteur pipette) and gently placed on 7 mL percoll (20% v/v, Sigma-Aldrich). After centrifugation (2800 rpm, 7 min), the percoll containing ganglia remnants was removed and the pellet resuspended in 1 mL complete F-12 medium. After centrifugation (2800 rpm, 3 min), the supernatant was removed leaving approximately 30–60 *μ*L of fluid. The cells were resuspended in the remaining fluid and approximately 10–20 *μ*L of the cell suspension was placed on each of three poly-d-lysine/laminin 12 mm coverslips (Becton Dickinson CDC). The coverslips were placed at 37°C for two hours to allow the cells to adhere to the coverslips and afterwards, 2.5 mL of complete F-12 medium was added to each well. The cells were kept at 37°C until use (2–12 hours).

### 2.3. Calcium Imaging

The cells were transferred to PSS, adjusted to pH 7.4 and loaded with the fluorescent Ca^2+^-indicator Fura-2 acetoxymethyl ester (Fura-2/AM, 4.4 *μ*M) dissolved in DMSO and Chremaphor EL detergent (4 : 1) for 30–60 min at 37°C. After loading, the cells were transferred to fresh PSS buffer and allowed to stand for approx. 30 min in order to ensure completion of the cytoplasmatic dye deesterification [24].

Changes in intracellular calcium concentrations in neurons from C_8_-T_5_ DRG's were measured by FURA-2 ratiometric imaging in the acutely dissociated culture. At the time of measurement, cells were round and without processes.

The cells were illuminated alternately with light of 340 nm (specific for Ca^2+^-bound Fura-2) and 380 nm (specific for Ca^2+^-free Fura-2), and light of 510 nm was collected via a cooled CCD camera (Hamamatsu). The photomultiplier was coupled to a computer for data acquisition.

### 2.4. Experimental Protocols

Six different protocols were employed in the study, only one protocol per coverslip. Each coverslip was initially stimulated twice with 20 mM K^+^ for 10 s separated by 7 min wash with PSS. The first depolarization was undertaken to ensure vitality of the neurons and saturation of the intracellular compartments and was excluded from further analysis. After washout of the second administration of K^+^, neurons were stimulated for 10 s with one of the following solutions: (1) 0.3 *μ*M capsaicin, (2) 0.5 *μ*M capsaicin, (3) mixture of algogenic chemicals (AC) combined with 0.3 *μ*M capsaicin, (4) AC combined with 0.5 *μ*M capsaicin, (5) AC combined with 0.3 *μ*M capsaicin at pH 6.0, or (6) AC combined with 0.5 *μ*M capsaicin at pH 6.0. After stimulation with one of these solutions, the neurons were washed with PSS for 8 min. The experiment was concluded with a third K^+^ stimulation to evaluate the continued viability of the neuron for the extent of the protocol. The mixture of algogenic chemicals contained bradykinin, serotonin, prostaglandin E_2_, all in the concentration of 10^−5 ^M, and adenosine (10^−3 ^M). All substances used for stimulation were obtained from Sigma-Aldrich.

pH 6.0 was chosen for the two acidic protocols studies as DRG neurons containing TRPV1 and acid sensing ion channels (ASICs) have been demonstrated to react to this pH [25–27].

### 2.5. Analysis

Prior to experiments, inclusion criteria for data analysis were defined: (1) labeling with Fura-2, (2) only neurons included, (3) neurons should stay within its marked area throughout the experiments, (4) cells responding to 20 mM K^+^ were included (≥20% increase from baseline), and (5) only neurons returning to baseline during washout period were included. 

To monitor the [Ca^2+^]_*i*_ changes in single cells, specific areas of interest were chosen for cells and for background. For each time frame, the ratio of background-corrected fluorescence intensities detected at 510 nm and resulting from excitation at 340 and 380 nm was calculated:

(1) $$\begin{array}{c} R\;=\frac{I_{340}-I_{340}\left(\text{background}\right)}{I_{380}-I_{380}\left(\text{background}\right)}. \end{array}$$

This ratio is an arbitrary value and was used as a measure of the activity level and to record changes in the activity level of the neurons.

Baseline, peak value, and AUC of light intensity ratios were measured for each response in Chart 5 (AD Instruments). For K^+^ stimulations, AUC was calculated from 0 s to 180 s after initiation of response (AUC_0−180 s_); for all other stimulations, AUC_0−60 s_ was calculated, where 0 s indicates beginning of rise in [Ca^2+^]_*i*_. The maximum increase from baseline, that is, the peak height, was calculated for all neurons, both in absolute terms (Δ), and relative as the percentage increase from baseline (%-Δ). For all stimulations except K^+^, Δ, %-Δ and AUC were furthermore compared to the K^+^ response by calculating the ratio of a given response and the preceding K^+^ response for each neuron.

Neuron diameter was measured and the neurons were classified as small (S, <30 *μ*m), medium (M, 30–39 *μ*m), or large (L, ≥40 *μ*m), similar to the classification by Lu et al. [28]. To further analyze the distribution of neuron diameter in control and diabetic animals, the neurons were divided into bins, each of 5 *μ*m width.

Cells responding to 20 mM K^+^ were included in the analysis as neurons [18]. Responsiveness was defined as ≥ 20% increase from baseline (i.e., %-Δ ≥ 20%), after stimulation with any substance in the protocol.

During analysis it was found that neurons stimulated with 0.3 and 0.5 *μ*M Capsaicin elicited similar responses, and this was also seen for neurons stimulated with the combination of AC and either 0.3 or 0.5 *μ*M Capsaicin. For subsequent analysis, 0.3 and 0.5 *μ*M Capsaicin was therefore combined and henceforward is referred to as “Capsaicin” and furthermore, the protocols of AC in combination with either 0.3 or 0.5 *μ*M Capsaicin were combined and henceforward are referred to “AC + Capsaicin.”

### 2.6. Statistical Analysis

The results are presented either as mean ± SEM or as median (5th percentile; 95th percentile), depending on normality of data. Comparisons were made with Student's *t*-test or, when appropriate, the non-parametric Mann-Whitney Rank Sum Test. Comparisons of diameter distribution were done using Chi-square test. Statistical tests were performed with SigmaStat 3.11 and GraphPad Prism 5. *P* < 0.05 was used as level of significance.

## 3. Results

### 3.1. Animals

In total, 8 diabetic ZDF animals and 8 age-matched control ZDF rats were used in this study. At the age of 7 weeks, the ZDF diabetic rats had developed significantly higher blood glucose concentrations than control rats and this hyperglycemia persisted for the whole experimental period. At the time of experiment, all diabetic animals had an elevated blood glucose level of 27.5 ± 4.5 mM compared to 6.2 ± 0.5 mM in control animals (*P* < 0.001, *n* = 16). The body weight was larger in diabetic animals from 7 weeks of age and this increased body weight persisted throughout week 14 after which body weight no longer differed between control and diabetic animals. At the time of experiment, body weight was 378.0  ±  26.3 g for the diabetic animals and 365 ± 23.9 g for the control animals (*P* > 0.05, *n* = 16).

### 3.2. Neurons

The mean cell diameter of the recorded DRG neurons was slightly smaller for diabetic than for control animals (25 *μ*m (18.0; 33.2) versus 26 *μ*m (19.0; 33.0), *P* < 0.05, *n* = 1124). 

**Figure 1:**
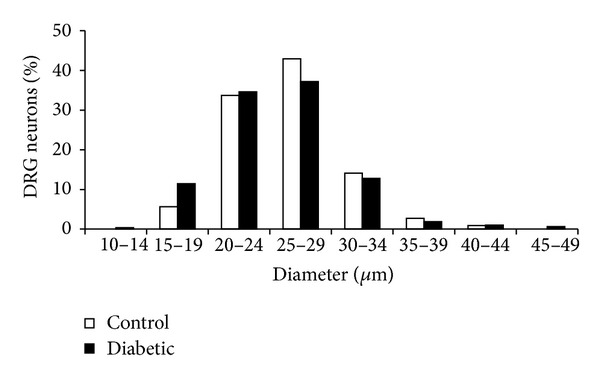
Distribution of neuronal cell diameter. The neurons were divided into 5 *μ*m bins. Small-diameter neurons had diameters < 30 *μ*m, medium-sized neurons had diameters between 30–39 *μ*m, and large-diameter neurons had diameters of ≥ 40 *μ*m. The majority of tested neurons were small-sized. *n* (control) = 552, *n* (diabetic) = 586.

The distribution of small-, medium-, and large-sized neurons, though, did not differ between the two animal groups; for neurons from diabetic animals, 82% were small-sized, 17% medium-sized, and 1% large-sized compared to 83%, 15%, and 2%, respectively, for neurons from control animals. The distribution based on neuron diameter was also not significantly different in control and diabetic animals (Figure 1). When differentiating, however, between neurons responding to either of the capsaicin protocols (capsaicin-responsive) and neurons not responding to the tested capsaicin protocol (capsaicin-unresponsive), analysis showed that the distribution of “capsaicin-responsive” neurons from diabetic animals was significantly different from the distribution of control animals (*P* < 0.05) with a median neuron diameter in diabetic animals of 23 *μ*m (16.7; 30.0) compared to 25 *μ*m (18.9; 34.1) in control animals (*P* < 0.01).

**Figure 2:**
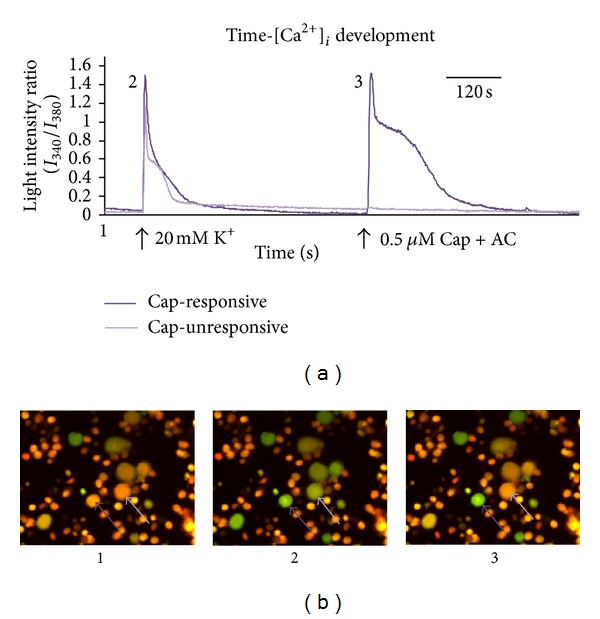
Example of calcium activity measurement. The figure depicts the trace of two cells obtained during a single experiment: a capsaicin-responsive (dark purple) and a capsaicin-unresponsive (light purple) neuron. The three small photomicrographs show the same neurons, at different stages of the experiment: (1) before stimulation, (2) during stimulation with 20 mM K^+^, and (3) during stimulation with 0.5 *μ*M Capsaicin + algogenic chemicals. Black arrows beneath the trace indicate the beginning of stimulation. Time scale bar; 120 s. Cap; capsaicin.

The baseline Ca^2+^ level did not differ between diabetic and control animals (0.58 (0.46; 0.80) versus 0.59 (0.47; 0.77), *P* > 0.05, *n* = 1127). Response to a given stimulus was elicited by an increase in intracellular Ca^2+^-level and viewed as an increase in fluorescence intensity ratio, that is, a peak that slowly returned to baseline. An example of a capsaicin-responsive and-unresponsive neuron following stimulation with 20 mM K^+^ and a capsaicin-containing stimulus is depicted in Figure 2. Neuron responses with and without shoulder were found among both groups of animals as well as among capsaicin-responsive and -unresponsive neurons and thus, presence or lack of shoulder in the response was not confined to single groups of neurons investigated.

### 3.3. K^+^-Induced Depolarization

**Figure 3:**
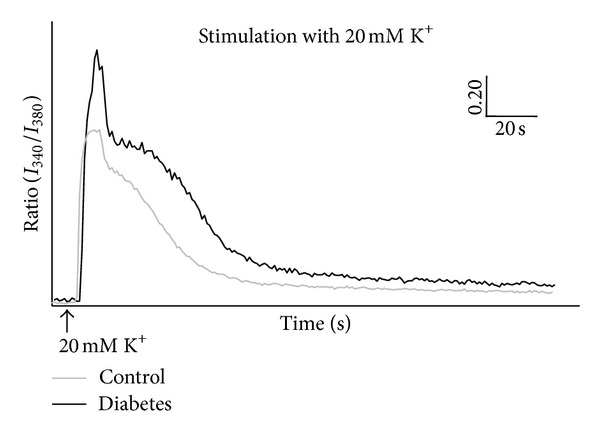
Example of time-[Ca^2+^]_*i*_ ratio development in dorsal root ganglion neurons from control and diabetic rats. The neurons were stimulated with 20 mM K^+^. For clarity in the illustration, the traces were background corrected. Arbitrary scale; vertical scale: 0.20 (340/380 nm light intensity ratio), and horizontal scale: 20 s.

Control as well as diabetic neurons responded to stimulation with 20 mM K^+^ by an increase in [Ca^2+^]_*i*_, assessed by an increase in 340/380 nm fluorescence ratio. The intracellular Ca^2+^ level returned to baseline during the washing period. A representative trace of the time-[Ca^2+^]_*i*_ relationship is shown in Figure 3. The diabetic neurons showed an overall increased responsiveness to stimulation with 20 mM K^+^ compared to neurons from control animals (Table 1). This was seen as an augmented AUC, peak height (Δ), and % increase from baseline (%-Δ). The increased responsiveness was observed both in “capsaicin-responsive” and “capsaicin-unresponsive” neurons (Table 2). For both control and diabetic animals it was seen that capsaicin-responsive neurons generally had a higher responsiveness to 20 mM K^+^ than capsaicin-unresponsive neurons (Table 2).

### 3.4. Capsaicin Protocols

**Figure 4:**
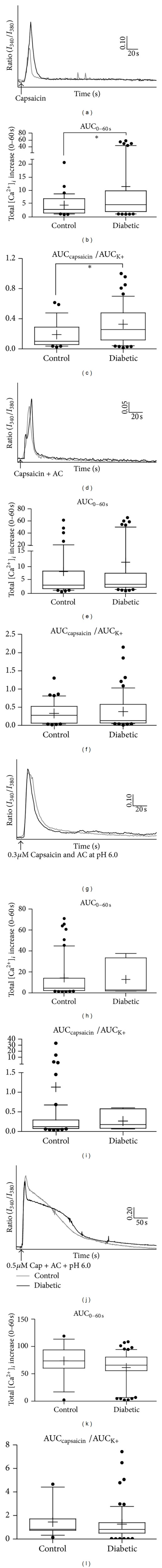
Traces and box plots from capsaicin experiments. Panels (a), (d), (g), and (j) show traces (examples) of the time-[Ca^2+^]_*i*_ development for control and diabetic dorsal root ganglion neurons. The arrows indicate the onset of 10 sec stimulation with (a) capsaicin, (d) combination of Capsaicin and algogenic chemicals (AC); (g) combination of 0.3 *μ*M Capsaicin and AC at pH 6.0, and (j) 0.5 *μ*M Capsaicin and AC at pH 6.0. The traces were background corrected. As a measure of [Ca^2+^]_*i*_, the vertical axis is shown as the 340/380 nm light intensity ratio. (a) Vertical scale: 0.10; horizontal scale: 20 s, (d) vertical scale: 0.20; horizontal scale: 20 s.; (g) vertical scale: 0.10; horizontal scale: 20 s., and (j) vertical scale: 0.20; horizontal scale: 20 sec. Panels (b), (e), (h), and (k) display box plots on the absolute area under the curve (AUC) derived from stimulation with the in (a), (d), (g), and (j) mentioned capsaicin-containing solutions. Panels (c), (f), (i), and (l) display box plots with median, 25th and 75th percentiles on the relative AUC of the capsaicin-containing solution-derived [Ca^2+^]_*i*_ increase and the preceding 20 mM K^+^-derived [Ca^2+^]_*i*_ increase. The error bars indicate the 10th and 90th percentiles. Values below the 10th and above the 90th percentiles are shown as dots. + indicates the mean. *P < 0.05.

Stimulation with capsaicin alone, 0.3 or 0.5 *μ*M (no combination) caused a rise in [Ca^2+^]_i_ for approximately 23% of the neurons in both groups of animals. The neurons from diabetic animals exhibited an augmented response, assessed by a larger peak height, percentage increase from baseline, and AUC (Figures 4(a)–4(c) and Table 3).

Capsaicin-responsive neurons stimulated with capsaicin in combination with the algogenic substances at either pH 7.4 or 6.0 were followed by increases in [Ca^2+^]_*i*_ in neurons from both diabetic and control animals (Figures 4(d), 4(g), and 4(j)). None of the stimulations gave rise to differences in peak height or AUC between diabetic and control animals (Figures 4(d)–4(k), Table 3).

**Figure 5:**
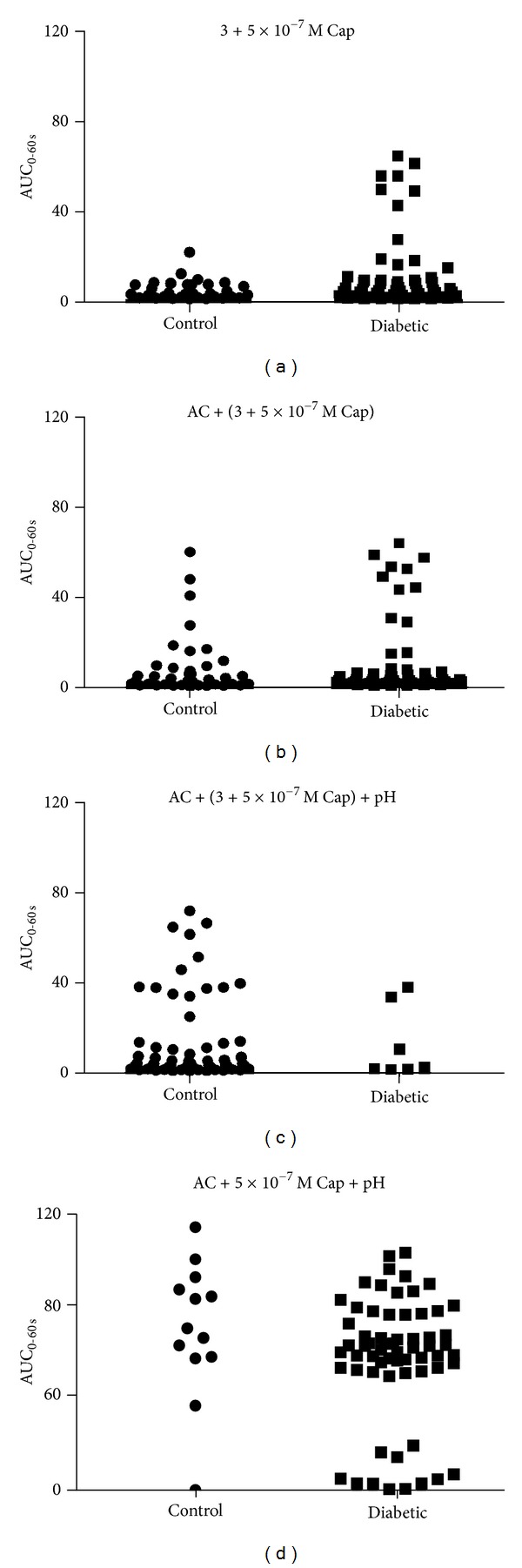
Scatter plots displaying AUC for neurons from control and diabetic animals derived from stimulation with (a) capsaicin, (b) combination of Capsaicin and algogenic chemicals (AC); (c) combination of 0.3 *μ*M Capsaicin and AC at pH 6.0, and (d) 0.5 *μ*M Capsaicin and AC at pH 6.0.

Scatter plots of the capsaicin responses indicated that neurons responding to capsaicin-containing solutions might be divided in high- and low-responders (Figure 5).

Stimulation with 0.5 *μ*M capsaicin in combination with the algogenic chemicals at pH 6.0 caused a significantly augmented Ca^2+^ response compared to any of the other stimulations. This was seen for neurons from diabetic animals (Δ; 1.63 (0.16; 1.87), AUC; 63.51 (2.87; 97.95), *P* < 0.001 compared to every other stimulation of diabetic neurons, *n* = 63) as well as from control animals (Δ; 1.7 (0.21; 2.37); AUC; 72.13 (1.58; 117.24), *P* < 0.001 compared to every other stimulation of control neurons, *n* = 13). The responses were similar in neurons from control and diabetic animals (*P* > 0.05).

## 4. Discussion

The major findings of the present study were the following: (1) baseline [Ca^2+^]_*i*_ were unaltered in C_8_-T_5_ DRG neurons from ZDF diabetic compared to control animals, (2) K^+^-induced and (3) capsaicin-induced depolarization caused augmented [Ca^2+^]_*i*_ currents in neurons from diabetic animals.

### 4.1. Baseline Ca^2+^-Levels

The current study showed no differences in baseline [Ca^2+^]_*i*_ in C_8_-T_5_ DRG neurons from diabetic and control animals. This is in accordance with several other studies performed on DRG neurons from STZ-diabetic mice and rats, db/db diabetic mice and BB/W rats [18, 29–32], and on dorsal horn neurons from lumbar L_6_–L_7_ spinal cord segments from STZ-diabetic rats [19, 33]. Shutov et al. [34], however, have found an increased baseline [Ca^2+^]_*i*_ in L_4_–L_6_ DRG neurons from STZ-induced diabetic rats at the same time as an unaltered baseline in DH neurons, suggesting a differential effect by diabetes on primary and secondary neurons [34]. The segmental location of the neurons investigated also appear to be affected differently by diabetes as neurons with longer axons are more susceptible to neuropathic changes than neurons with shorter axons [8]. This is supported by reports of an increase in baseline [Ca^2+^]_i_ in STZ-diabetic DRG neurons extracted from L_4_–L_6_ levels where the neurons have longer axons because they innervate the hind paws [18, 34], while Huang and colleagues, in the same study, found no baseline changes in C_5_-L_3_ DRG neurons [18], which agrees with the results from the present study on C_8_-T_5_ DRG neurons.

### 4.2. K^+^-Induced Depolarization

The present study found an increased depolarization-induced [Ca^2+^]_*i*_ current in DRG neurons from ZDF diabetic animals compared to control animals. The increase comprised an absolute and a percentage increase in amplitude from baseline [Ca^2+^]_*i*_ and an increased AUC_(0−180 s)_. Hall et al. also found an increased elevation of [Ca^2+^]_*i*_ in thoracic DRG neurons (d. 20–40 *μ*m) after stimulation with 50 mM KCl for 6 s in BB/W rats [32]. Most other studies with DRG and DH neurons have not detected differences in the amplitude of [Ca^2+^]_*i*_ elevation between diabetic and nondiabetic rats and mice [19, 29–31, 33–35]. An exception, however, is Huang et al. who found a decreased amplitude of depolarization-induced [Ca^2+^]_*i*_ current in small DRG neurons from L_4_–L_6_ in STZ-diabetic animals (14-week duration) [18]. The decrease in amplitude which contrasts the findings of the present study could be explained by the increase in [Ca^2+^]_*i*_ baseline that Huang et al. found in the DRG neurons from diabetic animals [18], which might have limited the potential change in [Ca^2+^]_*i*_ in these neurons. The discrepancy between studies might also be related to the duration of diabetes since Huang et al. investigated neurons from STZ-diabetic rats with longer duration (8–14 weeks) of diabetes than what was seen in other studies. The type of diabetes might also influence calcium homeostasis differently, since the present study and the study by Hall et al., both of which found increases in amplitude were performed in ZDF and BB/W rats [32], respectively compared to the other studies in which STZ-diabetic animals were employed [18, 19, 29–31, 33–35].

There is a general agreement on a prolonged recovery following depolarization-induced [Ca^2+^]_*i*_ currents in neurons from diabetic rats and mice, measured as recovery time, residual [Ca^2+^]_*i*_ (60 or 90 s after depolarization), or, as in the present study, AUC_(0−180 s)_ [18, 19, 29–31, 34, 35]. This suggests that the diabetes-induced effect on recovery of [Ca^2+^]_*i*_ is somewhat fundamental as the studies are based on both type 1 and type 2 diabetes, different rodent species, neuron sizes, segmental orientations of the neurons investigated, and on different parts of the nervous system (DRG or DH). The level of [Ca^2+^]_*i*_ in the DRG neurons is the sum of mechanisms leading to Ca^2+^-entry into the intracellular space and mechanisms leading to removal of Ca^2+^ from the intracellular space [36]. During stimulation, intracellular calcium level rises because calcium enters the cell via plasmalemmal voltage- and ligand-gated, and store-operated Ca^2+^-channels, and also because calcium is released from the endoplasmatic reticulum [36, 37]. Kinetics of the recovery phase is affected by release of Ca^2+^ ions from the mitochondria. 

Voltage-dependent calcium currents through L- and N-channels are enhanced in dorsal horn neurons of STZ rats *in vitro* [33]. This is in accordance with the study by Hall et al. who found a decreased inhibitory effect of the opioid agonist Dyn A in thoracic DRGs from BB/W diabetic rats [32] and an impaired inhibitory G-protein function in thoracic and lumbar DRGs from BB/W and STZ rats, thus contributing to an increased calcium influx [38].

Several mechanisms thus seem to contribute to the increased K^+^-induced depolarization found in the present study, and since these changes are seen in different species, diabetes models, and at different DRG levels, the impact of diabetes on neuronal calcium metabolism seems to be universal.

### 4.3. Capsaicin Protocols

The present study showed an increased elevation of [Ca^2+^]_*i*_ following stimulation with capsaicin (pooled results for 0.3 and 0.5 *μ*M) and this agrees with studies by Hall et al. and Hong and Wiley who found an increased peak [Ca^2+^]_*i*_ in thoracic DRG neurons (20–40 *μ*m) from BB/W rats and larger and longer-lasting inward currents in DRG neurons from STZ-diabetic rats, respectively, following application of 1 *μ*M Capsaicin [32].

The expression of TRPV1 has been shown to be decreased in the hearts of STZ-diabetic mice [39] although Strecker and colleagues reported unaltered capsaicin-induced CGRP release from human right atrium from subjects with diabetes [40].

The findings of increased Ca^2+^-response to K^+^ and capsaicin show that diabetes causes functional changes in these neurons. The augmented response measured in neurons from diabetic animals following stimulation with capsaicin suggests that the function or expression of TRPV1 might be altered by diabetes. Song et al. have found a decreased expression of membrane TVPR1 in heart [41] which is supported by Hong and Wiley who found decreased TRPV1 expression in small L_1_–L_6_ DRG neurons [42]. They showed, however, in the same study that functional TRPV1 was increased in neurons from diabetic animals and furthermore, the acid-evoked (pH 5.0) transient, mediated by TRPV1, was increased in these neurons [42] which is in accordance with the increased capsaicin response seen in the present study.

The function of the PKC-induced phosphorylation, and thus sensitization, of TRPV1 [43] has been shown to be increased in lumbar DRG neurons from STZ diabetic rats [42].

Glutamate receptors (GluR) colocalized with TRPV1 on small diameter DRG neurons have been demonstrated to contribute to the activity of the capsaicin-induced TRPV1 sensitization [44] and GluR could possibly contribute to the finding in the present study that capsaicin-responsive neurons elicited larger Ca^2+^ responses than neurons from capsaicin-unresponsive neurons (the difference was seen both in diabetic as well as control neurons).

Scatter plots of the responses to capsaicin-containing solutions indicated that the tested neurons might be either high- or low-responders. It cannot be ruled out that diabetes has a differentiated effect on these two potentially different subpopulations of neurons.

When 0.5 *μ*M capsaicin was applied in combination with AC at acidic pH 6.0, the calcium response was larger (amplitude and AUC) for neurons, regardless of diabetic state, compared to all other applied solutions. This is in accordance with other studies showing that the capsaicin-induced neurons activity can be amplified by lowering pH and by adding inflammatory substances, such as bradykinin 5-HT, PGE_2_, and adenosine [10, 13, 26, 27]. There was no difference, however, between neurons from control and diabetic animals.

## 5. Conclusion

The findings of increased Ca^2+^-response to K^+^ and capsaicin show that diabetes causes functional changes in the investigated DRG neurons. The augmented response measured in neurons from diabetic animals following stimulation with capsaicin suggests that the function or expression of TRPV1 might be altered which is supported by other studies.

This is, to the best of our knowledge, the first study to investigate calcium activity in DRGs from ZDF rats and the study shows that the diabetes-induced effects on peripheral neuron function have common characteristics with other diabetic animal models. The ZDF rat thus seems to be a relevant model for studying diabetes-induced neuronal alterations in DRG neurons.

Diabetic neuropathy is a very complex condition with multiple factors playing part in the pathophysiology. The present study suggests that diabetes induces changes in DRG neuron function and this might be a contributing factor to the altered pain perception and the altered neuronal response by cardiac neurons seen in diabetic patients during myocardial ischemia and infarction.

## Figures and Tables

**Table 1:** Quantitative response of all neurons to 10 s stimulation with 20 mM K^+^. Results are shown as median (5%–95% percentile).

Animal	*n*	AUC_0–180 s_	Δ	%-Δ
Control	549	30.00 (3.04–110.11)	0.92 (0.25–1.47)	156.36 (41.77–248.25)
Diabetic	578	41.41 (3.89–121.72)*	1.08 (0.31–1.72)*	182.26 (35.86–308.52)*

AUC: Area under the curve for 0–180 s, Δ: the absolute change in [Ca^2+^]_*i*_ (fluorescence ratio), %-Δ: the change in [Ca^2+^]_*i*_ (fluorescence ratio) measured in % change from baseline.*P < 0.001 versus control neurons.

**Table 2:** Quantitative response to 10 s stimulation with 20 mM K^+^. The results were subcategorized according to whether they responded to the capsaicin protocol the respective neuron was stimulated with. Results are shown as median (5%–95% percentile).

Animal	Capsaicin response	*n*	AUC_0–180 s_	Δ	%-Δ
Control	Y	157	34.68 (3.05–138.27)	1.05 (0.27–1.65)	175.36 (39.50–290.96)
	N	392	**28.02 (3.02–93.19)** ^#^	** 0.88 (0.25–1.30)** ^#^	** 149.64 (42.24–233.10)** ^#^
Diabetic	Y	193	42.97 (5.08–155.09)	1.25 (0.38–1.96)*	202.78 (60.60–353.75)*
	N	385	**40.99 (3.45–105.17)** ^∗$^	**1.01 (0.28–1.59)** ^∗#^	**170.37 (21.97–288.22)** ^∗#^

AUC: Area under the curve for 0–180 s, Δ: the absolute change in [Ca^2+^]_*i*_ (fluorescence ratio), %-Δ: the change in [Ca^2+^]_*i*_ (fluorescence ratio) measured in % change from baseline. Y: Yes and N: No. *P < 0.001 versus corresponding control neurons, ^#^ *P* < 0.001 and ^$^ *P* < 0.05 versus corresponding capsaicin-responsive neurons.

**Table 3:** Results from stimulation with capsaicin-containing solutions. Results are shown as Median (5th percentile; 95th percentile).

Animal	Stimulus	*n*	Δ	%-Δ	AUC_0–60 s_	AUC_cap_/AUC_K^+^_	Δcap/ΔK^+^	%-Δ_cap_/%-Δ_K^+^_
Control	0.3/0.5 *μ*M Cap	32	0.22 (0.12; 0.88)	31.13 (19.85; 148.10)	2.94 (0.98; 15.09)	0.10 (0.03; 0.61)	0.23 (0.09; 0.68)	0.23 (0.09; 0.71)
	0.3/0.5 *μ*M Cap + AC	45	0.21 (0.11; 1.34)	35.29 (20.89; 250.89)	2.88 (0.72; 48.31)	0.23 (0.01; 0.83)	0.42 (0.08; 0.99)	0.44 (0.08; 1.11)
	0.3 *μ*M Cap + AC + pH 6.0	60	0.42 (0.12; 1.64)	73.81 (22.70; 289.41)	4.10 (0.85; 64.82)	0.09 (0.01; 4.56)	0.44 (0.12; 1.66)	0.42 (0.11; 1.74)
	0.5 *μ*M Cap + AC + pH 6.0	13	1.7 (0.21; 2.37)	240.67 (30.89; 352.63)	72.13 (1.58; 117.24)	0.83 (0.12; 4.75)	1.35 (0.25; 2.39)	1.47 (0.25; 2.30)

Diabetic	0.3/0.5 *μ*M Cap	55	0.41 (0.12; 1.50)*	**61.54 (21.88; 218.25)** ^ #^	4.83 (1.13; 55.17)*	0.26 (0.03; 0.89)*	0.42 (0.07; 1.12)	0.46 (0.07; 1.14)*
	0.3/0.5 *μ*M Cap + AC	58	0.27 (0.11; 1.53)	47.62 (20.81; 294.64)	3.22 (0.90; 60.81)	0.12 (0.03; 1.32)	0.28 (0.13; 1.16)	0.29 (0.13; 1.20
	0.3 *μ*M Cap + AC + pH 6.0	7	0.23 (0.11; 1.55)	35.09 (24.44; 249.06)	2.34 (1.33; 38.01)	0.14 (0.03; 0.56)	0.21 (0.08; 1.04)	0.23 (0.08; 1.06)
	0.5 *μ*M Cap + AC + pH 6.0	63	1.63 (0.16; 1.87)	276.27 (22.75; 414.13)	63.51 (2.87; 97.95)	0.84 (0.02; 5.13)	1.24 (0.11; 1.93)	1.21 (0.11; 2.12)

Results are shown as AUC_0–60 s_, relative AUC (AUC_cap_/AUC_K^+^_), peak height (Δ), % increase from baseline (%-Δ), relative peak height relative to peak height of the preceding K^+^ response (Δcap/ΔK^+^ and %-Δ_cap_/%-Δ_K^+^_). *n*; number of cells, AUC; area under the curve; cap, capsaicin. *P < 0.05, ^#^ *P* < 0.01 compared to control neurons.
